# CTRP1 Attenuates Cerebral Ischemia/Reperfusion Injury via the PERK Signaling Pathway

**DOI:** 10.3389/fcell.2021.700854

**Published:** 2021-08-04

**Authors:** Huizhi Fei, Pu Xiang, Wen Luo, Xiaodan Tan, Chao Gu, Maozhu Liu, Mengyuan Chen, Qiong Wang, Junqing Yang

**Affiliations:** ^1^Key Laboratory of Biochemistry and Molecular Pharmacology, College of Pharmacy, Chongqing Medical University, Chongqing, China; ^2^Chongqing Three Gorges Medical College, Chongqing, China

**Keywords:** CTRP1, apoptosis, endoplasmic reticulum stress, cerebral ischemia reperfusion injury, PERK signaling pathway

## Abstract

Cerebral ischemic stroke is one of the leading causes of death worldwide. Previous studies have shown that circulating levels of CTRP1 are upregulated in patients with acute ischemic stroke. However, the function of CTRP1 in neurons remains unclear. The purpose of this study was to explore the role of CTRP1 in cerebral ischemia reperfusion injury (CIRI) and to elucidate the underlying mechanism. Middle cerebral artery occlusion/reperfusion (MCAO/R) and oxygen–glucose deprivation/reoxygenation (OGD/R) models were used to simulate cerebral ischemic stroke *in vivo* and *in vitro*, respectively. CTRP1 overexpression lentivirus and CTRP1 siRNA were used to observe the effect of CTRP1 expression, and the PERK selective activator CCT020312 was used to activate the PERK signaling pathway. We found the decreased expression of CTRP1 in the cortex of MCAO/R-treated rats and OGD/R-treated primary cortical neurons. CTRP1 overexpression attenuated CIRI, accompanied by the reduction of apoptosis and suppression of the PERK signaling pathway. Interference with CTRP1 expression *in vitro* aggravated apoptotic activity and increased the expression of proteins involved in the PERK signaling pathway. Moreover, activating the PERK signaling pathway abolished the protective effects of CTRP1 on neuron injury induced by CIRI *in vivo* and *in vitro*. In conclusion, CTRP1 protects against CIRI by reducing apoptosis and endoplasmic reticulum stress (ERS) through inhibiting the PERK-dependent signaling pathway, suggesting that CTRP1 plays a crucial role in the pathogenesis of CIRI.

## Introduction

Cerebral ischemic stroke is a serious cerebrovascular disease leading to serious neurological disability and death worldwide (Katan and Luft, 2018). Each year, about 795,000 people experience a new or recurrent stroke, with ischemic stroke accounting for approximately 87% of the cases (Virani et al., 2021). Thus, it is urgent to find effective methods and drugs for the treatment of cerebral ischemic stroke. Ischemic stroke is caused by stenosis or occlusion of the cerebral supplying artery and transient or permanent local reduction of cerebral blood flow. It has been shown that millions of neurons and billions of synapses are destroyed during each minute of ischemia in patients (Saver, 2006; Desai et al., 2019). Thus, the critical treatment of ischemic stroke in the clinical setting involves the timely restoration and improvement of cerebral blood supply. However, exacerbation of the tissue injury may occur with the restoration of blood supply, which is known as cerebral ischemia reperfusion injury (CIRI).

A variety of mechanisms have been proposed to be involved in CIRI, including oxidative stress, intracellular Ca^2+^ overloading, the endoplasmic reticulum stress (ERS), uncontrolled inflammatory response, energy metabolism disorders, and subsequent apoptosis. Studies have shown that neuron apoptosis has a key role in the pathogenesis of CIRI and neurological disability (Estaquier et al., 2012). It has been suggested that inhibition of apoptosis would alleviate CIRI (Candelario-Jalil, 2009; McCabe et al., 2018). Therefore, suppression of apoptosis is crucial in acute stroke therapy.

The endoplasmic reticulum is a critical organelle in maintaining the balance of cell survival and death. The ERS plays a dual role in cell apoptosis (Sano and Reed, 2013). On the one hand, ERS initiates the unfolded protein response (UPR) to maintain cell balance and protect cells from stress. On the other hand, if the stress persists or becomes severe, cell apoptosis is triggered via activation of caspase 12 and DNA damage-inducible transcript 3 protein (CHOP) (Ochoa et al., 2018). A number of studies have confirmed that apoptosis induced by ERS plays a crucial role in neuron death and neuron injury associated with CIRI (Nakka et al., 2010; Caldeira et al., 2014; Kalogeris et al., 2016; Wu et al., 2020) and provides the potential targets for future treatment of CIRI via inhibiting ERS (Zhao et al., 2018; Xu et al., 2019; Wang K. et al., 2020; Bai et al., 2021).

ERS initiates cell stress response by upregulating the expression of ERS chaperone GRP78. GRP78 is able to control the activation of three transmembrane ERS sensors, including protein kinase RNA-like endoplasmic reticulum kinase (PERK), activating transcription factor 6 (ATF6), and inositol-requiring enzyme 1 (IRE1), which transduce UPR and subsequently trigger apoptosis through a binding-release mechanism (Walter and Ron, 2011; Glembotski et al., 2020). CHOP is most characteristic of and the key signaling pathway involved in ERS-related apoptosis (Li et al., 2014; Hu et al., 2019; Oyadomari and Mori, 2019). Previous studies have reported that PERK is required for the expression of the CHOP gene (Harding et al., 2000) because PERK^–/–^ and activating transcription factor 4 (ATF4)^–/–^ cells failed to induce CHOP expression during ERS (Harding et al., 2003). According to previous studies, both *in vitro* and *in vivo*, the PERK/ATF4/CHOP signaling pathway plays a pivotal role in apoptosis and cell death induced by ERS (Harding et al., 2000; Cao et al., 2012; Chen et al., 2014; Liu et al., 2016; Wei et al., 2019).

The PERK signaling pathway is activated after ischemia and reperfusion, and the expressions of eukaryotic initiation factor 2α (eIF2α), p-eIF2α, PERK, p-PERK, ATF4, caspase 12, and cleaved-caspase 3 are increased by cerebral ischemic/reperfusion (Owen et al., 2005; Hu et al., 2017; Liu et al., 2018). It has been shown that the inhibition of PERK-CHOP-mediated apoptosis protected against CIRI (Hu et al., 2017; Liu et al., 2018; Li Y. et al., 2020).

C1q/TNF-related protein1 (CTRP1) is a secreted protein mostly expressed in adipose tissues with major functions in glucolipid metabolism, inflammation, cell proliferation, and apoptosis. CTRP1 plays an important role in glucolipid metabolism. The level of CTRP1 in patients with type 2 diabetes is significantly increased (Pan et al., 2014; Han et al., 2016a; Yagmur et al., 2019). CTRP1 significantly improves insulin resistance (Xin et al., 2017), enhances fatty acid oxidation (Peterson et al., 2012), and protects against diet-induced hyperglycemia (Han et al., 2016b). Loss of CTRP1 disrupted glucose and lipid homeostasis (Rodriguez et al., 2016). Previous studies suggested that CTRP1 could be considered as a diagnostic biomarker and an oncogene in human glioblastoma (Chen and Su, 2019). CTRP1 links macrophage lipid metabolism to inflammation and atherosclerosis (Van Hinsbergh and Eringa, 2016; Wang X. Q. et al., 2016). CTRP1 has also been shown to play an important role in Kawasaki disease (Feng et al., 2018; Weng et al., 2020) and atherosclerosis (Lu et al., 2016). Recent studies have shown that CTRP1 exhibits potential protective effects on renal disease (Barbieri et al., 2018; Li W. et al., 2020; Rodriguez et al., 2020) and that it is involved in the pathogenesis of cardiovascular diseases. The level of CTRP1 in serum is increased in patients with coronary artery diseases (Shen et al., 2014; Tang et al., 2015; Wang H. et al., 2016), hypertension (Jeon et al., 2008; Su et al., 2019), and congestive heart failure (Yang et al., 2017). CTRP1 inhibits cardiac hypertrophy and fibrosis via activation of the AMPKα pathway (Wu L. et al., 2018). CTRP1 protects against DOX-induced cardiac injury via inhibition of cell apoptosis (Chen et al., 2018). CTRP1 deficiency exacerbates myocardial infarct size, cardiomyocyte apoptosis, and proinflammatory gene expression induced by ischemia reperfusion injury (Yuasa et al., 2016). These studies have indicated that CTRP1 might play a potentially important role in cerebral ischemic stroke. In addition, a recent paper reported that the circulating level of CTRP1 was increased in patients with acute ischemic stroke, and CTRP1 attenuated microglia autophagy and inflammatory response by regulating the Akt/mTOR pathway (Wang H. et al., 2020). However, there have been no reports on the effects of CTRP1 on neurons subjected to ischemic injury.

In the present study, we established cerebral ischemic/reperfusion model *in vivo* and *in vitro* to explore the physiological/pathophysiological role of CTRP1 in neuron apoptosis and ERS. We reported a protective role of CTRP1 against CIRI *in vivo* and *in vitro* via the PERK/ATF4/CHOP signaling pathway.

## Materials and Methods

### Animals, Reagents, and Ischemia Models

Animal experiments were reviewed and approved by the Animal Care and Use Committee of Chongqing Medical University. Adult male Sprague–Dawley rats were purchased and kept at the Animal Center of Chongqing Medical University. All rats had a body weight of 260 ± 20 g. The groups used in the study are shown in Supplementary Figures 1A,B.

Ischemia/reperfusion injury was produced by middle cerebral artery occlusion/reperfusion (MCAO/R) as previously reported (Peng et al., 2020). The rats were anesthetized by intraperitoneal injection of 4% chloral hydrate (0.1 mg/ml). The right common carotid artery (CCA), the external carotid artery (ECA), and the internal carotid artery (ICA) were exposed carefully. A nylon suture was inserted into the CCA for MCAO for 90 min, then the suture was withdrawn for further reperfusion. In the sham group, the surgical operation was performed in the same way but without production of MCAO. The cerebral blood flow (CBF) was examined in ischemia by a laser Doppler flowmeter (PeriFlux system 500; Perimed, Beijing, China). A decrease of 70% was considered successful (regional CBF < 70% of the baseline, as shown in Supplementary Figure 1C). The rats were sacrificed, and the brain and blood were quickly collected at 24 h after MCAO/R operation.

The CTRP1 overexpression lentivirus was provided by JiKai (Shanghai, China). The CTRP1 siRNA was designed and synthesized by Abclonal (Wuhan, China). CCT020312 was provided by MCE (Shanghai, China).

### Neurological Deficiency Assessment

The neurological dysfunction of rats was measured at 24 h after MCAO/R using Zea–Longa neurological deficit scores (Longa et al., 1989). The neurological scores follow a five-point scale: 0: no neurological deficiency; 1: failing to extent the right forelimb; 2: circling to the contralateral side; 3: leaning to the injured said; 4: lack of spontaneous motor activity and presence of a depressed level of consciousness.

### 2,3,5-Triphenyltetrazolium Chloride (TTC) Staining

The entire brains were quickly removed and frozen at −20°C for 20 min and sliced into 2-mm-thick sections. The sections were stained with 2% TTC (Sigma-Aldrich, St. Louis, MO, United States) at 37°C for 20 min, and fixed in 4% paraformaldehyde at 4°C. The brain sections were photographed and measured in Image J software (NIH, Bethesda, MD, United States). The percentage infarct volume was calculated as: (area of ischemic tissue in the ischemic hemisphere)/area of all tissue × 100%.

### Histopathological Analysis

The rats treated with MCAO/R were anesthetized and perfused with PBS and 4% paraformaldehyde. The brains were cut into 5-μm-thick sections after being dehydrated in a graded series of alcohols and embedded in paraffin. The sections were stained with hematoxylin-eosin (HE) reagents for histopathological examination. For HE staining, we stained the sections with hematoxylin for 5 min and separated the color in 1% hydrochloric acid alcohol for 25 s; sections were backed to blue for 30 s with 1% ammonia, stained with eosin for 5 min, and finally dehydrated with increasing concentrations of alcohol and hyalinized with dimethyl benzene.

### Immunofluorescence Analysis

The brain sections were dewaxed in dimethylbenzene and put in a microwave oven for antigen retrieval. The sections were treated with a blocking buffer (5% BSA with 1% Trion X-100 in PBS) for 1 h at 37°C, then incubated with anti-CTRP1 (20 μg/ml, NBP1-76626, Novus; 1:10, sc-81943; Santa Cruz Biotechnology, Inc., Sta. Cruz, CA, United States), anti-Neuron (1:200, GTX30773, Genetex), anti-Iba1 (1:200, DF6442, Affinity Biosciences, Jiangsu, China), and anti-GFAP (1:200, DF6040, Affinity) overnight at 4°C. The sections were incubated with fluorescein-labeled secondary antibodies for 1 h. An antifade mounting medium containing DAPI was used to cover the sections. Images were observed with a fluorescence microscope (Nikon, Tokyo, Japan).

The immunofluorescence in neurons was detected in the same way.

### Primary Cortical Neuron Culture and OGD/R Exposure

Primary cortical neurons were isolated from fetal rats as previously reported (Bailey and Lahiri, 2006). The cells were cultured with neurobasal medium (Gibco; Thermo Fisher Scientific, Waltham, MA, United States) supplemented with 2% B27 (Gibco, United States) and 1% glutamine (Gibco, United States) in an incubator with 5% CO_2_ at 37°C. The medium was half-replaced every 3 days. The neurons were used to establish oxygen and a glucose deprivation/reoxygenation (OGD/R) model after 7 days. Primary neurons were cultured in a glucose-free DMEM medium (Gibco, United States) in an atmosphere of 5% CO_2_ and 1% oxygen and 94% N_2_ for half an hour. After OGD operation, the cells were cultured in original medium for 24 h at 37°C in a humidified incubator with 5% CO_2_.

### Cell Transfection and Drug Intervention

Lentivirus CTRP1 (LV-CTRP1) and control vector (LV-NC) were transfected in accordance with the instructions. CTRP1 siRNA (si-CTRP1) and control siRNA (si-NC) were transfected in accordance with the instructions. The efficiency of overexpression was measured by qRT-PCR. CCT020312 was used to activate the PERK signaling pathway for 24 h before OGD/R.

### 3-(4,5-Dimethyl-Thiazol-2-yl)-2,5-Diphenyl-Tetrazolium (MTT) Assay

Neurons were cultured in 24-well plates at a density of 30 × 10^5^ cells/ml and treated as described above. Next, 40 μl MTT (5 mg/ml; Sigma-Aldrich) was added into each well and incubated at 37°C for 4 h. Then, 200 μl dimethyl sulfoxide was added to dissolve the formazan in the dark. The optical density was determined at 490 nm in a multifunction microplate reader (Thermo Fisher Scientific).

### Western Blot Analysis

Total protein was extracted from the cortex and neurons by a RIPA lysis buffer containing 1% phenylmethane sulfonyl fluoride and 1% phosphatase inhibitor. The protein content was detected by a BCA Protein Assay Kit (Dingguo Changsheng Biotechnology Co., Ltd., Beijing, China). Western blotting was performed as described previously, and protein bands were developed by an ECL detection system. The following primary antibodies were purchased: anti-CTRP1 (1:1,000, 12209-1-AP; Proteintech, Wuhan, China), anti-β-actin (1:5,000, 20536-1-AP; Proteintech), anti-PERK (1:1,000, AF5304; Affinity), anti-phospho-PERK (Ser555) (1:1,000, AF4499; Affinity), anti-GRP78 (1:1,000, AF53661; Affinity), anti-ATF4 (1:1,000, 10835-1-AP; Proteintech), Bax (1:10,000, 60267-1-AP; Proteintech), Bcl-2 (1:1,000, BF9103; Affinity), eIF2α (1:1,000, AF6087; Affinity), CHOP (1:1,000, AF5280; Affinity), ATF6 (1:1,000, AF6009; Affinity), IRE1α (1:1,000, DF7709; Affinity), phospho-IRE1α (ser724) (1:1,000, AF7150; Affinity), phospho-eIF2α (ser51) (1:1,000, AF3087; Affinity), cleave-caspase 3 (1:1,000, 9661; Cell Signaling Technology, Inc., Danvers, MA, United States), and caspase 12 (1:1,000, 55238-1-AP; Proteintech).

### Quantitative RT-PCR Analysis

Total RNA was isolated from whole blood, cortex, and primary neurons using a Trizol reagent (Vazyme Biotech, Jiangsu, China). Then, mRNA was subjected to reverse transcription using HiScript Q Select RT SuperMix (Vazyme). The reaction product was subjected to quantitative RT-PCR analysis via stepwise amplification performed based on the primer sequence templates. The primer sequences of β-actin for rats were as follows: forward, 5′-TGTCACCAACTGGGACGATA-3′; reverse, 5′-GGGGTGTTGAAGGTCTCAAA-3′. The CTRP1 primers for rats were as follows: forward, 5′-CTGAGCCTTGTGC CACGAGTTC-3′; reverse: 5′-TCACGCAGGCTA GGGCTA TACC-3′.

### TUNEL Assay

TUNEL assay was performed to detect neuronal apoptosis in line with the instruction of a TUNEL Cell Apoptosis Detection Kit (Servicebio, Hubei, China).

Paraffin-embedded sections were routinely dewaxed by xylene for 1 h, then washed with PBS and incubated in 1%Triton-100 at room temperature for 20 min. Then, the sections were incubated with a 50 μl equilibration buffer for 30 min and immersed in a 56 μl TUNEL mixture (1 μl recombinant TdT enzyme, 5 μl TMR-5-dTTP labeling mix and 50 μl equilibration buffer) for 90 min at 37°C in a dark place. Finally, the sections were incubated in 2 μg/ml DAPI for 10 min. A fluorescence microscope (Nikon) was used to examine the samples. The apoptosis in primary neurons was detected using the same methods.

### ELISA Assay

ELISA kits (Dingguo) were used to determine the concentration of mediators of CTRP1 in the blood of MCAO/R rats.

### Co-immunoprecipitation (COIP) Assay

CTRP1 antibody (1:50) and GRP78 (1:80) were mixed with protein A/G beads (MCE) at 4°C overnight. The beads were mixed with an appropriate amount of protein at 4°C overnight then washed with PBST and separated with the immunoprecipitates using SDS-PAGE. Western blot was used to analyze the immunoprecipitates.

### Statistical Analysis

Statistical analysis was performed in GraphPad Prism 7 (GraphPad software, United States). All data were expressed as mean ± SEM. Statistical significance was determined by *t*-test for two-group comparisons or one-way analysis of variance (ANOVA) for multiple-group comparisons. A *p*-value below 0.05 indicated statistical significance.

## Results

### The Expression of CTRP1 Significantly Decreased in the Cortex and Increased in the Serum of MCAO/R-Treated Rats

**FIGURE 1:**
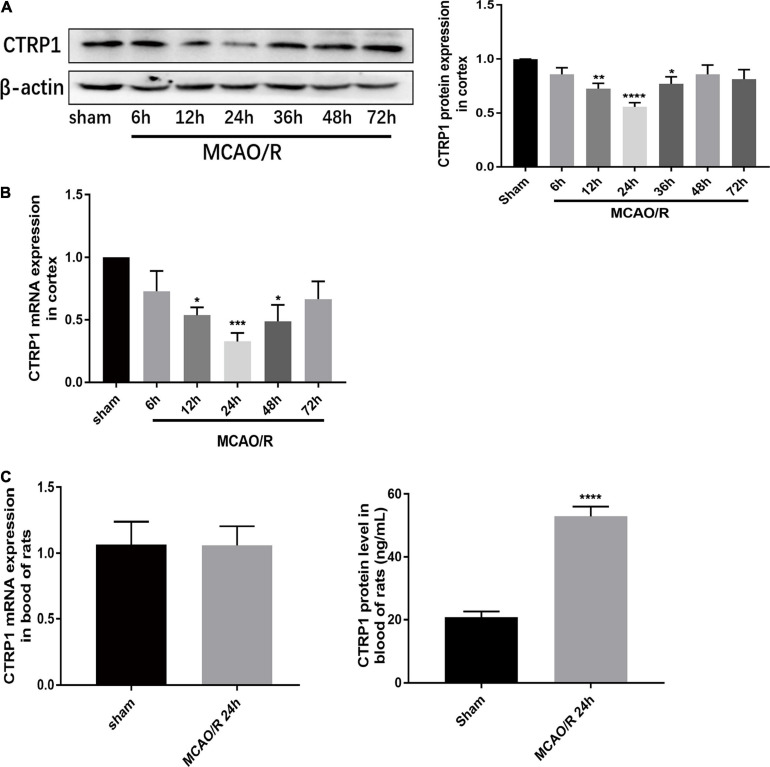
Relative expression of CTRP1 in MCAO/R-treated rats. **(A)** Relative protein expression of CTRP1 in the cortex detected by Western blot, *n* = 4 per group. **(B)** Relative mRNA expression of CTRP1 at different times after MCAO/R in the cortex, *n* = 4 per group. **(C)** Relative mRNA expression and concentration of CTRP1 in the serum of MCAO/R rats detected by qRT-PCR and ELISA, *n* = 12 per group. ****p < 0.0001, ***p < 0.001, **p < 0.001, *p < 0.05 vs. sham group.

To investigate the expression of CTRP1 in the cortex and blood of MCAO/R- treated rats, we examined the mRNA and protein expression levels by qRT-PCR, Western blot, and ELASA kits, respectively. As shown in Figures 1A,B, compared with the sham group, the CTRP1 mRNA and protein expression levels significantly decreased and presented the lowest level at 24 h after reperfusion in the cortex. Interestingly, the protein level of CTRP1 was significantly increased in blood, while mRNA level of CTRP1 did not (Figure 1C).

### The Expression of CTRP1 Was Mostly Located at Neurons in the Brain

**FIGURE 2:**
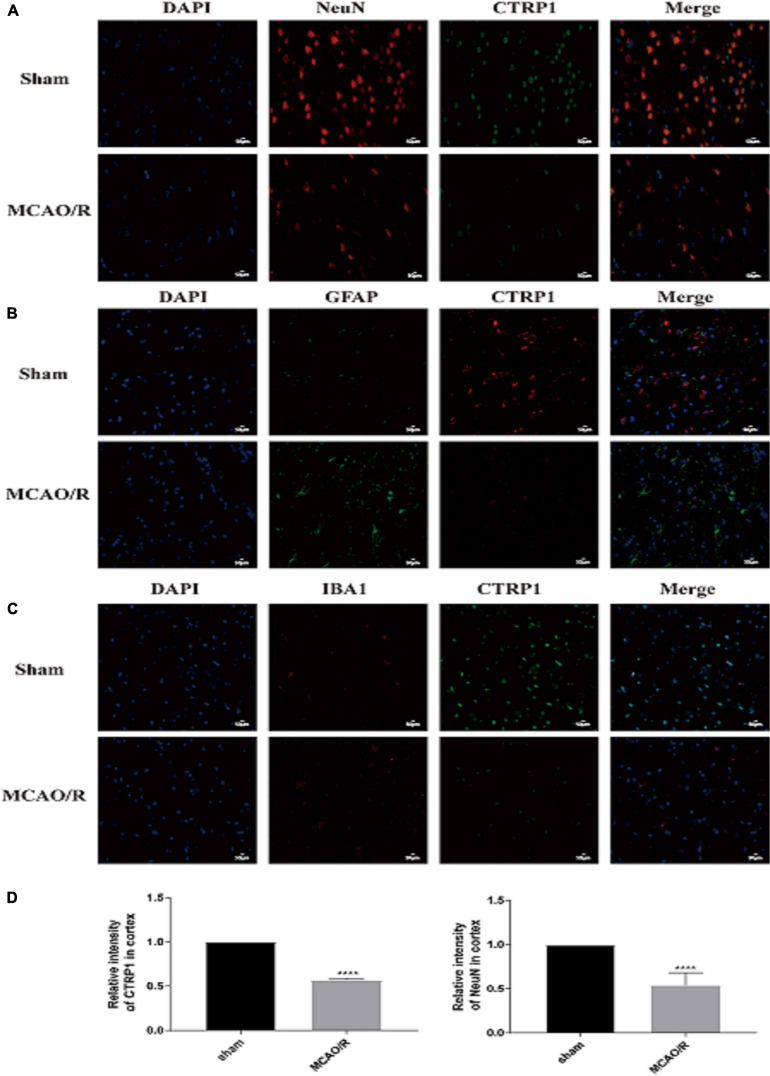
The location and expression of CTRP1 in the brain analyzed by immunofluorescence (*n* = 3). **(A)** The expression of CTRP1 in neuron in the cortex, NeuN was used to label neuron. CTRP1 expression was observed by fluorescence microscope and is shown by green fluorescence. NeuN expression is shown by red fluorescence. The nuclei were stained with DAPI and is shown by blue fluorescence. **(B)** The expression of CTRP1 in astroglia in the cortex. GFAP was used to label astroglia. CTRP1 expression is shown by red fluorescence. GFAP expression is shown by green fluorescence. **(C)** The expression of CTRP1 in microglia in the cortex. IBA1 was used to label microglia. CTRP1 expression is shown by green fluorescence. IBA1 expression is shown by red fluorescence. **(D)** The intensity of CTRP1 and NeuN in the cortex. The representative images were acquired under × 400 magnification, scale bars = 50 μm. ****p < 0.0001 vs. sham group.

To identify the expression and location of CTRP1 in the brain tissue of MCAO/R-treated rats, we detected CTRP1 expression by immunofluorescence. The results showed that CTRP1 was widely expressed in the brain tissue. Most CTRP1-positive cells were co-located with NeuN positive cells (Figure 2A), a small amount was co-located with IBA1 (Figure 2C) and hardly co-located with GFAP (Figure 2B). This finding indicated that CTRP1 was mostly expressed in neurons, a small amount in microglia, and hardly in astrocyte. Compared with the sham group, the CTRP1- and NeuN-positive cells and the intensity of positivity decreased significantly (Figure 2D), and the morphology of astrocytes and microglia was changed after MCAO/R.

### Overexpression of CTRP1 Attenuated Brain Injury in MCAO/R-Treated Rats

**FIGURE 3:**
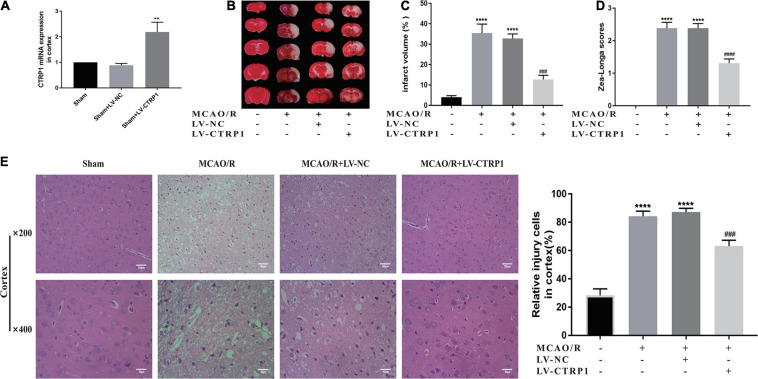
CTRP1 attenuated brain injury in MCAO/R-treated rats. **(A)** The infection efficiency of LV-CTRP1 in the cortex. *n* = 3 per group. **(B,C)** Representative images of TTC staining and quantitative analysis of infarct volume. *n* = 4 per group. **(D)** Zea-longe scores. *n* = 13 per group. **(E)** HE staining and relative positive cells in the cortex. *n* = 3 per group. The representative images were acquired under × 400 magnification, scale bars = 50 μm, × 200 magnification, scale bars = 100 μm. ****p < 0.0001, vs. sham group, **p < 0.01 vs. sham + LV-NC group, ^####^ *p* < 0.0001, ^###^ *p* < 0.001 vs. MCAO/R + LV-NC group.

To determine whether CTRP1 had an effect on CIRI, CTRP1 overexpression lentivirus was given by intracerebroventricular injection, which significantly increased the mRNA expression of CTRP1 in the cortex (Figure 3A).

TTC assay and Zea-Longa scores were used to detect the brain infarct volume and behavior changes. HE staining was used to investigate the histopathological changes in the brain. Representative images of TTC and HE are shown in Figure 3.

Evidently, the infarct volume and neurological scores significantly increased in the MCAO/R group and the vehicle-treated MCAO/R group. There was no significant difference between the MCAO/R group and the vehicle-treated MCAO/R group. Compared with the vehicle-treated MCAO/R group, the administration of CTRP1 lentivirus significantly decreased the infarct volume and neurological scores (Figures 3B–D).

As shown in Figure 3E, compared with the sham group, the nerve cells in the MCAO/R and the vehicle-treated MCAO/R group presented significant pyknosis, broken or even disappeared or nuclear, and the brain tissue section presented severe brain injuries; after treatment with CTRP1, the nuclei were more complete, the number of nerve cells with nuclear pyknosis or broken was significantly reduced, and brain injuries in the brain tissue were overtly alleviated. In short, CTRP1 had a protective effect on CIRI.

### Overexpression of CTRP1 Alleviated Neuron Injury and Apoptosis in the Cortex of MCAO/R-Treated Rats

**FIGURE 4:**
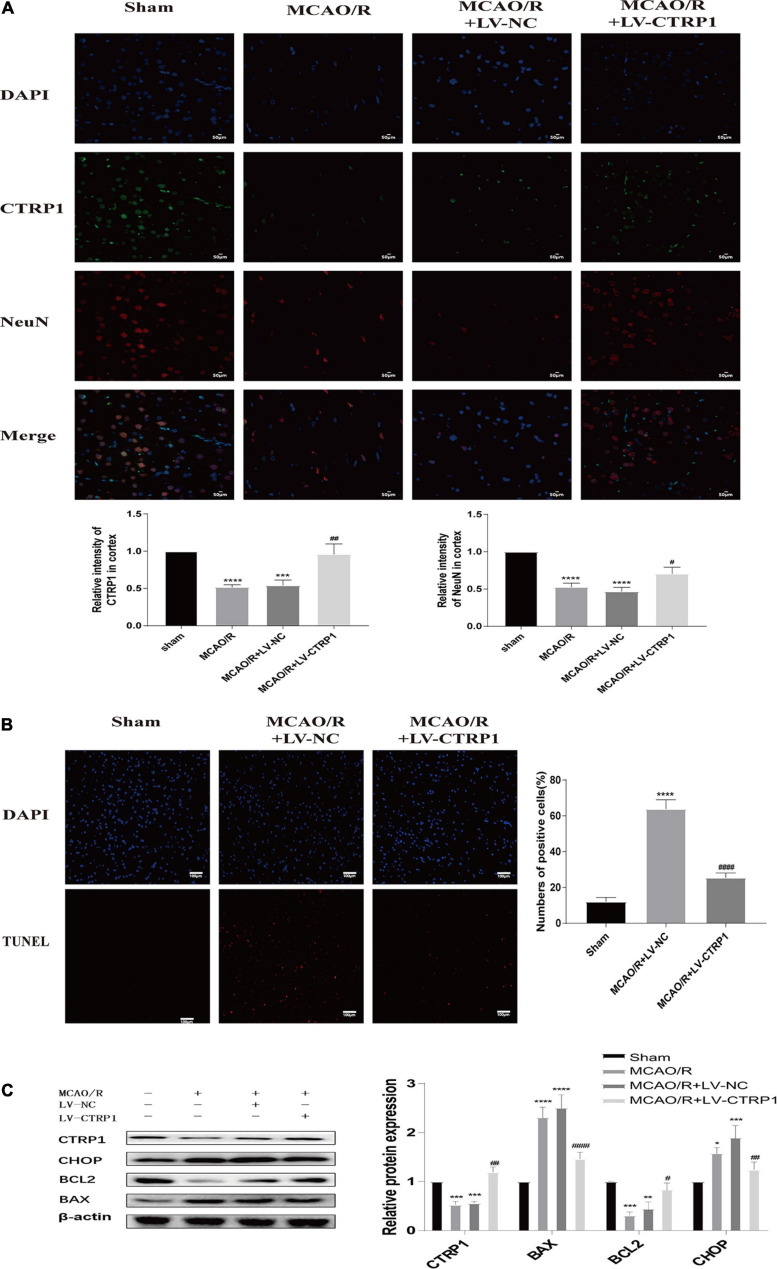
CTRP1 protected against cerebral ischemia reperfusion injury via alleviating neuron injury and apoptosis. **(A)** Neuron injury was analyzed by double labeling immunofluorescence staining. *n* = 3 per group. The representative images were acquired under × 400 magnification, scale bars = 50 μm. **(B)** Apoptosis in the cortex was analyzed by TUNEL, *n* = 3 per group. The representative images were acquired under × 200 magnification, scale bars = 100 μm. **(C)** Western blot analyzed the expression of CTRP1, BAX, Bcl-2, and CHOP in cortex. *n* = 4 per group. ****p < 0.0001, ***p < 0.01, **p < 0.01, *p < 0.05 vs. sham group; ^####^*p* < 0.0001, ^##^*p* < 0.01, ^#^*p* < 0.05 vs. MCAO/R + LV-NC group.

We found that the nerve cells were injured and lost in rats treated with MCAO/R, and CTRP1 alleviated the injury and loss of cells (Figure 3E). To identify whether CTRP1 protected against neuron injury in the cortex after MCAO/R, immunofluorescence was used to detect the CTRP1 and NeuN expression in the rat cortex. As shown in Figure 4A, the expression of CTRP1 and the intensity of positive NeuN cells were obviously reduced in rats treated with MCAO/R; CTRP1 overexpression markedly decreased the injury and loss of neuron in the cortex. The immunofluorescence results showed that CTRP1 overexpression increased the viability of neurons.

Given the close association between neuron death and apoptosis, we examined cell apoptosis in the cortex by TUNEL staining and Western blot. As shown in Figure 4B, compared with the sham group, abundant apoptotic cells were found in the cortex of rats in the vehicle-treated MCAO/R group; however, the number of apoptotic cells was significantly reduced after the administration of CTRP1. The TUNEL results suggested that CTRP1 attenuated apoptosis induced by MCAO/R. To further detect the effect of CTRP1 on apoptosis, we measured the expression of proteins associated with apoptosis (proapoptotic protein: BAX; antiapoptotic protein: Bcl-2; the marker of apoptosis induced by ERS: CHOP) by Western blotting. Compared with the sham group, the protein expression levels of BAX and CHOP significantly increased, and those of Bcl-2 decreased in the MCAO/R group and vehicle-treated MCAO/R group (Figure 4C). After CTRP1 overexpression treatment, the upregulation of CTRP1 resulted in the reduction of BAX and CHOP, as well as the increase of Bcl-2 level compared with the vehicle-treated MCAO/R group (Figure 4C). These data suggested that CTRP1 overexpression protected against MCAO/R-induced neuron injury via inhibition of cell apoptosis; moreover, inhibiting neuronal apoptosis induced by ERS might be involved in the mechanism of the CTRP1 protective effect on CIRI.

### CTRP1 Attenuated Cell Apoptosis via Inhibiting ERS Induced by MCAO/R

To further investigate whether ERS was involved in the effect of CTRP1 on apoptosis in MCAO/R-treated rats, the expression levels of ERS marker proteins (ATF6, ATF4, GRP78, IRE1α, p- IRE1α, PERK, p-PERK) were detected by Western blot.

**FIGURE 5:**
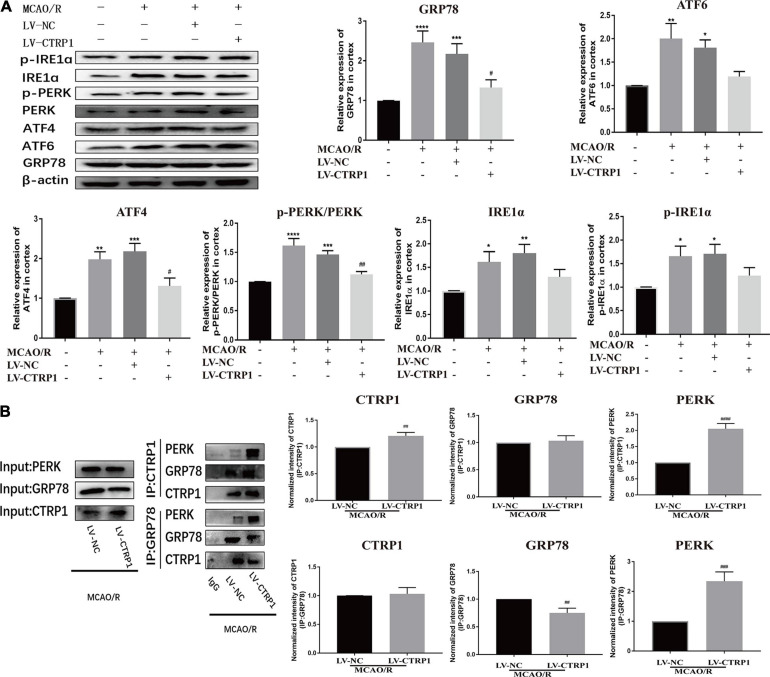
CTRP1 inhibited ERS via PERK signal pathway in the cortex of MCAO/R-treated rats. **(A)** Western blot analyzed the expression of PERK, p-PERK, GRP78, ATF6, ATF4, IRE1α, p-IRE1α. *n* = 4 per group. **(B)** CTRP1 affected the interaction between PERK and GRP78 after CIRI. *n* = 3 per group. ****p < 0.0001, ***p < 0.001, **p < 0.01, *p < 0.05 vs. sham group, ^####^*p* < 0.0001, ^###^*p* < 0.001, ^##^ *p* < 0.01, ^#^*p* < 0.05 vs. MCAO/R + LV-NC group.

As shown in Figure 5A, compared with the sham group, the relative protein expression level of GRP78, p-PERK/PERK, ATF4, ATF6, IRE1α, and p-IRE1α were significantly increased in the MCAO/R and vehicle-treated MCAO/R groups. CTRP1 overexpression significantly decreased the expression levels of GRP78 and ATF4 as well as the ratio of p-PERK/PERK. However, there was no significant difference in the expression of ATF6, IRE1α, and p-IRE1α between the CTRP1 overexpression group and the vehicle-treated MCAO/R group (Figure 5A). These results implied that the PERK/ATF4/CHOP signaling pathway in ERS was involved in the protective effect of CTRP1 on apoptosis.

To further understand the mechanisms by which CTRP1 may facilitate apoptosis in response to the PERK signaling pathway, we used COIP to analyze the interaction between CTRP1 and GRP78/PERK. We found an interaction between CTRP1 and GRP78 as well as an interaction between CTRP1and PERK as shown in Figure 5B. We noticed that the interaction between GRP78 and PERK was enhanced after CTRP1 overexpression treatment (Figure 5B). Interestingly, the interaction between CTRP1 and GRP78 was not changed, and the interaction between CTRP1 and PERK was significantly enhanced (Figure 5B). These results suggested that CTRP1 might have suppressed the dissolution between GRP78 and PERK to inhibit the PERK signaling pathway and alleviate PERK-associated apoptosis. In conclusion, these data implied that the PERK signaling pathway was involved in the underlying mechanism of CTRP1 effect.

### CTRP1 Protected Against Neuron Injury Induced by MCAO/R via Suppressing Apoptosis Through the PERK Signaling Pathway

To verify whether CTRP1 plays a protective role in CIRI through the PERK/ATF4/CHOP signaling pathway, we further injected the selective activator of PERK CCT020312 by intracerebroventricular injection.

**FIGURE 6:**
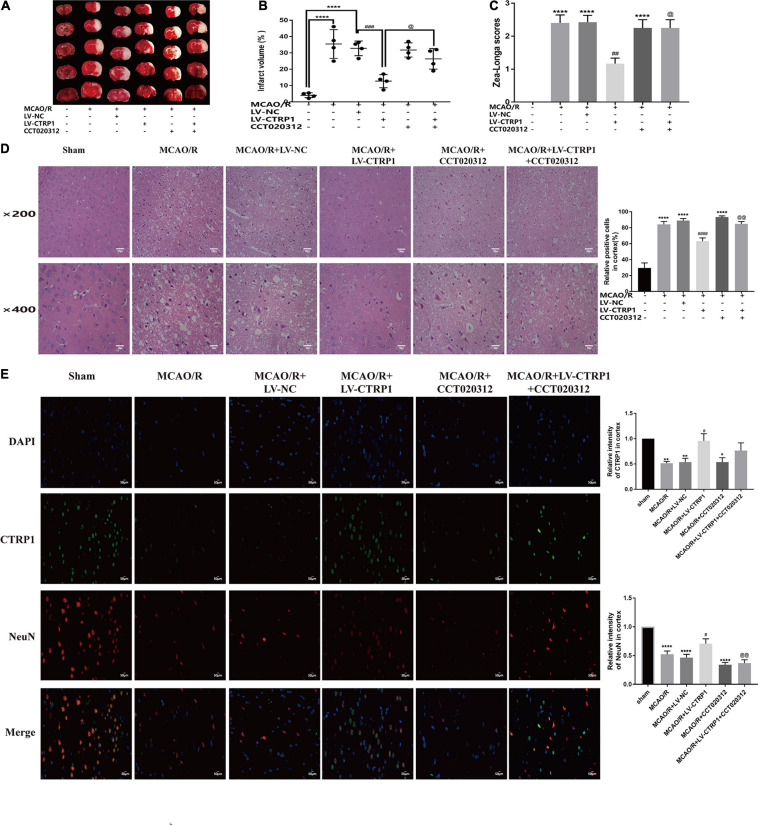
The protective effect of CTRP1 in CIRI was abolished by CCT020312. **(A,B)** TTC staining and infarct volume in the brain. *n* = 4 per group. **(C)** Zea-longe scores. *n* = 13 per group. **(D)** HE staining and relative positive cells in the cortex. *n* = 3 per group. The representative images were acquired under × 200 magnification, scale bars = 100 μm, × 400 magnification, scale bars = 50 μm. **(E)** Double labeling immunofluorescence staining in the cortex (× 400 magnification). *n* = 3 per group. The representative images were acquired under × 400 magnification, scale bars = 50 μm. ****p < 0.0001, ^∗∗^*p* < 0.01, *p < 0.05 vs. sham group, ^####^*p* < 0.0001, ^###^*p* < 0.001, ^##^*p* < 0.01, ^#^*p* < 0.05 vs. MCAO/R + LV-NC group; ^@@^*p* < 0.01, ^@^*p* < 0.05 vs. MCAO/R + LV-CTRP1 group.

As shown in Figure 6, CTRP1 overexpression significantly decreased the infarct volume and neurological deficit scores; however, these decreases were effectively reversed by CCT020312. CTRP1 overexpression alleviated cell damage and neuron injury induced by MCAO/R, but CCT020312 obviously blocked the protective effect of CTRP1 on neuron injury.

**FIGURE 7:**
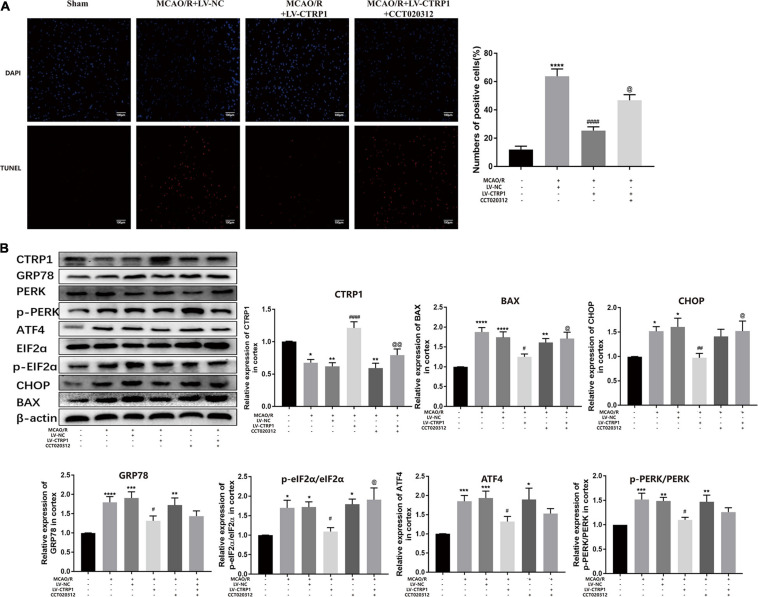
The function of CTRP1 on apoptosis and PERK signal pathway was inhibited by CCT020312. **(A)** Apoptosis was analyzed by TUNEL, *n* = 3 per group. The representative images were acquired under × 200 magnification, scale bars = 100 μm. **(B)** Western blot analyzed the expression CTRP1, BAX, CHOP, GRP78, ATF4, p-PERK, PERK, p-eIF2α, and eIF2α in the cortex. *n* = 6 per group. ****p < 0.0001, ***p < 0.001, **p < 0.01, *p < 0.05 vs. sham group, ^####^*p* < 0.0001, ^##^*p* < 0.01, ^#^*p* < 0.05 vs. MCAO/R + LV-NC group, ^@@^*p* < 0.01, ^@^*p* < 0.05 vs. MCAO/R + LV-CTRP1 group.

We further assessed neuron apoptosis by TUNEL. As shown in Figure 7A, the apoptosis of neurons was reduced in the CTRP1 overexpression group, whereas the reduction was reversed significantly by CCT020312. Then, we analyzed the protein expression of the PERK/ATF4/CHOP signaling pathway. Compared with those in the MCAO/R group, the expression levels of BAX, CHOP, GRP78, and ATF4 and the phosphorylation ratio of eIF2α and PERK showed a significant decline after CTRP1 upregulation (Figure 7B). The levels of BAX, CHOP, and the phosphorylation ratio of eIF2α were significantly upregulated after CCT0200312 treatment compared with the CTRP1 treatment group (Figure 7B). Interestingly, the expression levels of GRP78, AFF4, and the phosphorylation ratio of PERK in the MCAO/R + LV-CTRP1 + CCT020312 group were higher than those in the MCAO/R + LV-CTRP1 group, but there was no significant difference between the two groups. These data implied that the positive regulation of CTRP1 in MCAO/R was due to downregulation of apoptosis via the PERK signaling pathway.

### CTRP1 Increased Cell Survival by Suppressing Apoptosis Through the PERK Signaling Pathway in Primary Cortical Neurons After OGD/R

**FIGURE 8:**
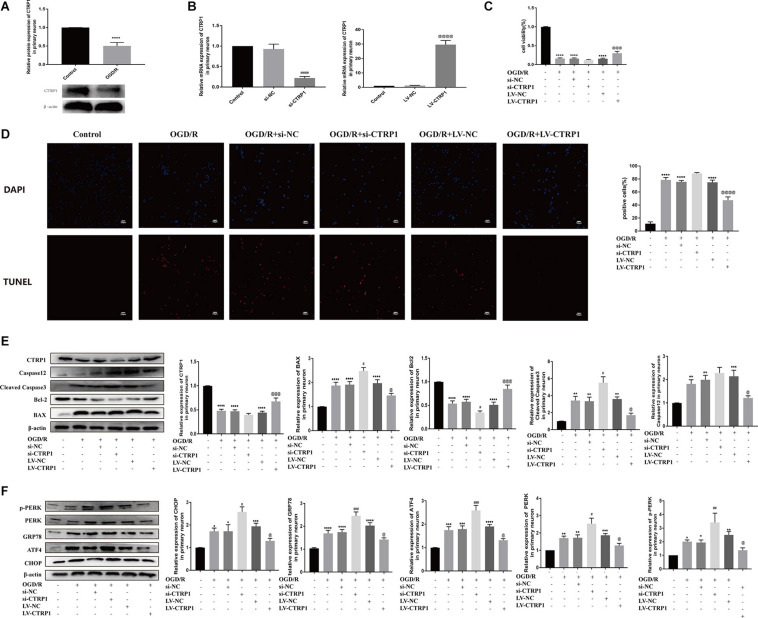
The function of CTRP1 in apoptosis and PERK signal pathway in neurons after OGD/R. **(A)** The expression of CTRP1 in neurons was analyzed by Western blot. n = 4 per group. **(B)** qRT-PCR analyzed the mRNA expression of CTRP1 after being transfected with siRNA and overexpression lentivirus, respectively. *n* = 3 per group. **(C)** Cell viability of neurons analyzed by MTT, *n* = 4 per group. **(D)** Apoptosis was analyzed by TUNEL, *n* = 3 per group. The representative images were acquired under × 200 magnification, scale bars = 100 μm. **(E)** The expression of CTRP1, BAX, Bcl-2, Cleaved caspase3, and Caspase 12 was analyzed by Western blot. *n* = 4 per group. **(F)** The protein expression of CHOP, GRP78, ATF4, PERK, and p-PERK in neurons. *n* = 4 per group. ****p < 0.0001, ***p < 0.001, **p < 0.01, *p < 0.05 vs. control group, ^####^*p* < 0.0001, ^###^*p* < 0.001, ^##^*p* < 0.01, ^#^*p* < 0.05 vs. OGD/R + si-NC group, ^@@@@^*p* < 0.0001, ^@@@^*p* < 0.001, ^@^*p* < 0.05 vs. OGD/R + LV-NC group.

Primary neurons treated by OGD/R were used to mimic cerebral ischemia/reperfusion injury *in vivo*. Our results showed that CTRP1 was highly expressed in neurons and was downregulated after OGD/R treatment, which was consistent with the results in rats (Supplementary Figure 2 and Figure 8A).

To explore the potential role of CTRP1 in response to OGD/R treatment, we transfected the neurons with the si-CTRP1 and CTRP1 overexpression lentivirus. qRT-PCR analysis confirmed that CTRP1 significantly decreased after si-CTRP1 transfection and increased after overexpression (Figure 8B). To determine the direct influence of CTRP1 expression on neurons after OGD/R, we used MTT analysis, TUNEL kits, and Western blot. As shown in Figures 8C,D, compared with the control group, cell viability and apoptosis were significantly altered in OGD/R group; at the same time, CTRP1 deficiency aggravated neuron loss and neuron apoptosis in neurons exposed to OGD/R, whereas CTRP1 overexpression led to a significant increase in cell viability and a decrease in neuron apoptosis. Western blot also revealed that the downregulation of CTRP1 resulted in the increased expression of BAX, and cleaved-caspase3 (apoptotic), as well as the decreased expression of Bcl-2 (anti-apoptotic); in contrast, the upregulation of CTRP1 inhibited the expression of apoptotic proteins and significantly elevated the level of anti-apoptosis proteins (Figure 8E). These data indicated that CTRP1 affected cell apoptosis. Further detection revealed that CTRP1 overexpression decreased GRP78, CHOP, ATF4, PERK, and p-PERK expression in OGD/R-treated neurons; however, GRP78, CHOP, ATF4, PERK, and p-PERK expression was raised in the case of CTRP1 deficiency (Figure 8F). Together, these data indicated that CTRP1 participated in the PERK/ATF4/CHOP signaling pathway-induced apoptosis in neurons exposed to OGD/R.

**FIGURE 9:**
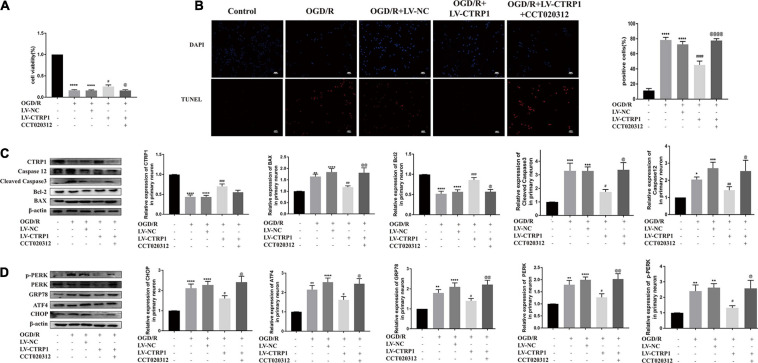
CCT020312 reversed the effect of CTRP1 on apoptosis and the PERK signal pathway after OGD/R. **(A)** MTT analyzed the cell viability in neurons. *n* = 4 per group. **(B)** TUNEL analyzed apoptosis in neurons. *n* = 3 per group. The representative images were acquired under × 200 magnification, scale bars = 100 μm. **(C)** The expression of CTRP1, BAX, Bcl-2, Cleaved caspase 3, Caspase 12 was analyzed by Western blot. *n* = 4 per group. **(D)** The relative expression of CHOP, GRP78, ATF4, PERK and p-PERK in neurons. *n* = 4 per group. ****p < 0.0001, ***p < 0.001, **p < 0.01, *p < 0.05 vs. control group, ^####^*p* < 0.0001, ^###^*p* < 0.001, ^##^*p* < 0.01, ^#^*p* < 0.05 vs. OGD/R + LV-NC group, ^@@@@^*p* < 0.0001, ^@@^ *p* < 0.01, ^@^ *p* < 0.05 vs. OGD/R + LV-CTRP1 group.

To confirm the hypothesis that the CTRP1 protection against OGD/R-induced injury depends on the PERK-CHOP pathway, the neurons were subjected to CCT020312 (a selective activator of PERK) before OGD/R. We found that CCT020312 effectively blocked the enhancing effect of CTRP1 on cell viability (Figure 9A). The number of apoptotic neurons examined by TUNEL decreased after CTRP1 overexpression, but the decrease was reversed by CCT020312 (Figure 9B). Furthermore, CTRP1 decreased the expression of BAX, Cleave-Caspase3, and Caspase 12, and increased the expression of Bcl-2; CCT020312 reversed these changes as well (Figure 9C). To evaluate whether CCT020312 blocked the effect of CTRP1 on the PERK signaling pathway, we measured the expression of proteins downstream of the PERK signaling pathway. Compared with vehicle group, CTRP1 downregulated the expression of CHOP, GRP78, ATF4, PERK, and p-PERK (Figure 9D). The combined treatment with CTRP1 and CCT020312 significantly increased the level of CHOP, GRP78, ATF4, PERK, and p-PERK (Figure 9D). These data clearly demonstrated that activated PERK significantly reversed the protective effects of CTRP1 on neurons.

## Discussion

To the best of our knowledge, this is the first report describing the protective role of CTRP1 in neuron injury induced by cerebral ischemia and reperfusion. In this study, CTRP1 overexpression decreased the infarct volume and neurological deficits, alleviated histopathological changes, and attenuated neuron injury and apoptosis by ERS via the PERK signaling pathway *in vivo*. Our *in vitro* experiments indicated that CTRP1 overexpression attenuated neuron injury and apoptosis via the PERK signaling pathway, while CTRP1 deficiency further aggravated neuron injury and apoptosis. Our *in vivo* and *in vitro* studies revealed a novel and crucial role of CTRP1 in preventing neuron injury induced by cerebral ischemia and reperfusion. Mechanistically, CTRP1 could bind with GRP78 and PERK, inhibit the dissociation between PERK and GRP78, and thus inhibited the PERK signaling pathway to prevent subsequent apoptosis and neuron injury. CTRP1 lost protection against neuron damage induced by cerebral ischemia and reperfusion after PERK activation.

CTRP1 is a secreted glycoprotein with major functions. Most studies explored the effect of CTRP1 on inflammation response in cardiomyocytes and microglia. A recent study revealed that CTRP1 was increased in patients with ischemic stroke and in OGD/R-treated BV2 cells (Wang H. et al., 2020). In our study, we first explored the co-location of CTRP1 in brain by immunofluorescence; we found that the expression of CTRP1 in brain tissue was mainly localized in neurons, a small extent in microglia, and hardly in astrocytes. Our *in vitro* study showed that CTRP1 was widely expressed in primary neurons and decreased after OGD/R treatment. The reason for the inconsistency of CTRP1 expression may be due to different responses to OGD/R in different cell types. Those results suggested the importance of studying the effect of CTRP1 in neurons.

In our studies, we also found that the mRNA and protein expression of CTRP1 significantly decreased in the cortex of rats treated with MCAO/R. Interestingly, the concentration of CTRP1 in the serum of MCAO/R was increased, while there was no significant difference in mRNA level between sham rats and MCAO/R-treated rats. These data suggested that the increased CTRP1 in serum may come from other tissues rather than blood itself, such as the brain and adipose tissue.

CTRP1 protein in peripheral blood might be derived from brain tissue because of the enhanced permeability of injured blood–brain barrier caused by CIRI. CTRP1 protein in the brain tissue might enter the blood circulation, resulting in the increase of CTRP1 protein in the blood and the decrease of CTRP1 content in the brain tissue. Studies have shown that CTRP3 could enter the cerebrospinal fluid (Schmid et al., 2017), but there have been no reports to prove that CTRP1 could pass the blood–brain barrier.

CTRP1 protein in peripheral blood might also be derived from adipose tissue. CTRP1 is likely an effective compensation for adiponectin deficiency. CTRP1 compensatorily upregulated in diabetic mice with reduced adiponectin (Wong et al., 2004; Davis and Scherer, 2008). Yuasa et al. (2016) found that CTRP1 upregulation might be a compensatory regulatory response to alleviate metabolic diseases and heart diseases. The level of adiponectin in blood is significantly reduced in patients and animal models of stroke (Ilhan et al., 2019; Yang et al., 2019). Therefore, we speculated that the elevated level of CTRP1 protein in blood might be a compensatory response to the decreased adiponectin level in the body, rather than originating from the blood cells themselves.

In addition, IL-1β and TNF-α promoted the secretion of CTRP1 in adipose tissue (Kim et al., 2006). The levels of inflammatory cytokines IL-1β, TNFα, and IL-6 in the blood of stroke patients and MCAO/R mice are increased (Chang et al., 2011; Lambertsen et al., 2012). These findings suggested that the elevated expression of CTRP1 protein in blood may be caused by the release of peripheral inflammatory factors induced by ischemia reperfusion and the promotion of CTRP1 secretion in adipose tissue. Similar results were reported in the study of Yang et al. (2017); CTRP1 protein expression was increased in the blood of patients with chronic heart failure, while CTRP1 mRNA and protein expression was increased in epicardial adipose tissue instead of cardiomyocytes. The possible mechanism was that the inflammatory state of patients stimulated the release of CTRP1 protein in epicardial adipose tissue, and CTRP1 protein entered the blood vessels in a paracrine way and was transported to the blood circulation.

These results suggested that CTRP1 was involved in the pathological process of CIRI. CTRP1 in peripheral blood might also be related to the pathological process, but its source, significance, and mechanism need to be further studied.

Considering the decreased CTRP1 expression, we investigated whether CTRP1 supplementation would prevent CIRI. As expected, CTRP1 overexpression protected the rats against MCAO/R-related infarct volume, neurological impairment, morphological changes, and neuron damage and apoptosis. CTRP1 overexpression also attenuated primary neuron injury and apoptosis induced by OGD/R, and CTRP1 deficiency further aggravated neuron injury and apoptosis. These results implied that CTRP1 played a pivotal protective role in CIRI.

Apoptosis is the key pathology of CIRI. It has been reported that the apoptosis in the penumbra could be paradoxically induced and aggravated by blood flow recanalization (Jennings, 2013). Thus, protecting neurons against delayed apoptosis in the penumbra is an indispensable strategy to improve post-stroke recovery. Previous studies have revealed the effect of CTRP1 on apoptosis. It has been reported that CTRP1 attenuated myocardial infarct size and cardiomyocyte apoptosis in acute ischemia injury, whereas lack of CTRP1 protein increased cardiomyocyte apoptosis and promoted myocardial injury (Yuasa et al., 2016). In mice with adriamycin-induced cardiac injury, CTRP1 attenuated apoptosis *in vivo* and increased the cell viability *in vitro* via the activation of AKT (Chen et al., 2018). In addition, lack of CTRP1 aggravated the apoptosis, while overexpression of CTRP1 inhibited apoptosis and oxidative stress injury (Jiang et al., 2020). These studies suggested that CTRP1 had a role in the positive regulation of cell apoptosis. In agreement with previous studies, we found that CTRP1 treatment attenuated neuron injury and apoptosis in MCAO/R rats. Our *in vitro* experiments showed that overexpression of CTRP1 improved cell viability and alleviated apoptotic activity, while CTRP1 deficiency accelerated neuron loss and apoptosis in OGD/R-treated primary neurons. These findings indicated that CTRP1 protected against neuron injury induced by cerebral ischemic reperfusion by inhibiting apoptosis.

We also found *in vivo* that CTRP1 reduced the level of GRP78, a biomarker of ERS, and the expression of CHOP, a key downstream factor of ERS-induced apoptosis, implying that ERS-induced apoptosis was involved in the protective function of CTRP1.

ERS plays important roles in neuronal injury and apoptosis after CIRI (Wu et al., 2017). ERS-related apoptosis is triggered and deteriorated in the reperfusion period and could last for 72 h (Wu F. et al., 2018). CHOP plays a critical role in the decision on the fate of the cells and could be a maker of ERS-related apoptosis (Hetz, 2012; Intemann et al., 2014). The ERS is initiated mainly through upregulation of GRP78. GRP78 is an ER chaperone that binds with PERK, ATF6, and IRE1α. PERK branch activation predominantly causes a rapid reduction in global rates of translation, while the IRE1 and ATF6 branch signaling induces a transcriptional response resulting in expression of chaperones and components of the protein degradation machinery (Hughes and Mallucci, 2019). PERK branch signaling has been reported to induce apoptosis under acute or prolonged ER stress (Gong et al., 2017; Wang et al., 2019). Our *in vivo* experiments showed that the expression levels of CHOP, GRP78, PERK, ATF6, ATF4, and IRE1α were increased after MCAO/R, while CTRP1 had a suppressing effect on the PERK branch and no significant effect on ATF6 and IRE1. We also found that CTRP1 overexpression suppressed the PERK signaling pathway, while CTRP1 deficiency further activated the PERK signaling pathway *in vitro*. These results implied that the PERK signaling pathway was involved in the protective effect of CTRP1.

Dissociation from GRP78 is the beginning of activation of the PERK signaling pathway. PERK autophosphorylated and then phosphorylated eIF2α, activating ATF4, resulting in the induction of the downstream gene CHOP (GADD153) (Harding et al., 2000, 2002; Luo et al., 2003). We used COIP to explore the effect of CTRP1 on the PERK signaling pathway. Our results showed that CTRP1 interacted with GRP78 and PERK. The CTRP1 supplement blocked the release of GRP78 and PERK; while the interaction between CTRP1 and GRP78 was not affected, the interaction between CTRP1 and PERK was enhanced. This may indicate that CTRP1 inhibits ERS, especially PERK signaling, by preventing the dissociation of PERK from GRP78, which results in the suppression of PERK signaling rather than the effect on interaction with GRP78. Similar to our findings, previous studies have shown that the weakened binding between GRP78 and PERK may change the threshold of ERS and promote ERS-related apoptosis (Chen et al., 2019).

Previous studies revealed that PERK was required for apoptosis triggered by ERS (Harding et al., 2003; Verfaillie et al., 2012). CCT020312 is a selective activator of PERK signaling (Stockwell et al., 2012; Bruch et al., 2017; Li X. et al., 2020). In the present study, CCT020312 was used to activate PERK to explore whether the effect of CTRP1 depended on the inhibition of PERK signaling. We found that the protective effects of CTRP1 on infarct size, neurological deficit, and apoptosis were reversed partly by the administration of CCT020312 *in vivo*. CCT020312 also reversed the effect of CTRP1 on neuron apoptosis and cell survival *in vitro*. These data suggested that the effect of CTRP1 on ERS was due to the suppression of PERK signaling, but not completely. In conclusion, our study indicated that ERS was involved in the protective action of CTRP1 on ischemia/reperfusion injury, especially the PERK signaling pathway in ERS.

In recent studies, PERK played a critical role in working memory and memory flexibility by regulating calcium dynamics in the brain and pancreatic beta cells, which share electrophysiological similarities with neurons (Wang et al., 2013; Zhu et al., 2016a,b; Sowers et al., 2018). Further studies are needed to understand whether the regulation of calcium dynamics is involved in the effect of CTRP1 on PERK.

In summary, we demonstrated that CTRP1 alleviated MCAO/R- and OGD/R-induced injury in neurons by inhibiting apoptosis through the PERK/ATF4/CHOP signaling pathway. Overexpression of CTRP1 may be a potential therapeutic approach for CIRI and improving brain function after ischemic stroke.

It is worth mentioning that our study has limitations. Even though we found that CTRP1 suppressed the dissociation of PERK and GRP78 to inhibit the PERK signaling, further studies need to explore the mechanism of CTRP1 action on GRP78/PERK dissociation and other underlying mechanisms in CIRI.

## Data Availability Statement

The original contributions presented in the study are included in the article/Supplementary Material, further inquiries can be directed to the corresponding author/s.

## Ethics Statement

The animal study was reviewed and approved by the Institutional Animal Ethics Committee of Chongqing Medical University, Chongqing, China.

## Author Contributions

JY and HF conceived and designed the study. HF, PX, WL, CG, XT, MC, ML, and QW were involved in the whole experiment. HF wrote the manuscript. All authors edited the manuscript and approved the submitted version.

## Conflict of Interest

The authors declare that the research was conducted in the absence of any commercial or financial relationships that could be construed as a potential conflict of interest.

## Publisher’s Note

All claims expressed in this article are solely those of the authors and do not necessarily represent those of their affiliated organizations, or those of the publisher, the editors and the reviewers. Any product that may be evaluated in this article, or claim that may be made by its manufacturer, is not guaranteed or endorsed by the publisher.

## Supplementary Material

Supplementary Figure 1 Experimental groups and protocol. **(A)** The groups in the studies. **(B)** Experimental protocol schedule. **(C)** Changes in cerebral blood flow were monitored during MCAO by laser Doppler flowmeter.

Supplementary Figure 2 The identification of neurons and the expression of CTRP1 in primary cortical neurons (*n* = 3). The representative images were acquired under × 100 magnification, scale bars = 200 μm, × 200 magnification, scale bars = 100 μm.
