# Supplementary figures and images for: CTRP1 Attenuates Cerebral Ischemia/Reperfusion Injury via the PERK Signaling Pathway
Source: Front Cell Dev Biol. 2021 Aug 4;9:700854. doi: 10.3389/fcell.2021.700854 (PMC8371340; doi:10.3389/fcell.2021.700854)

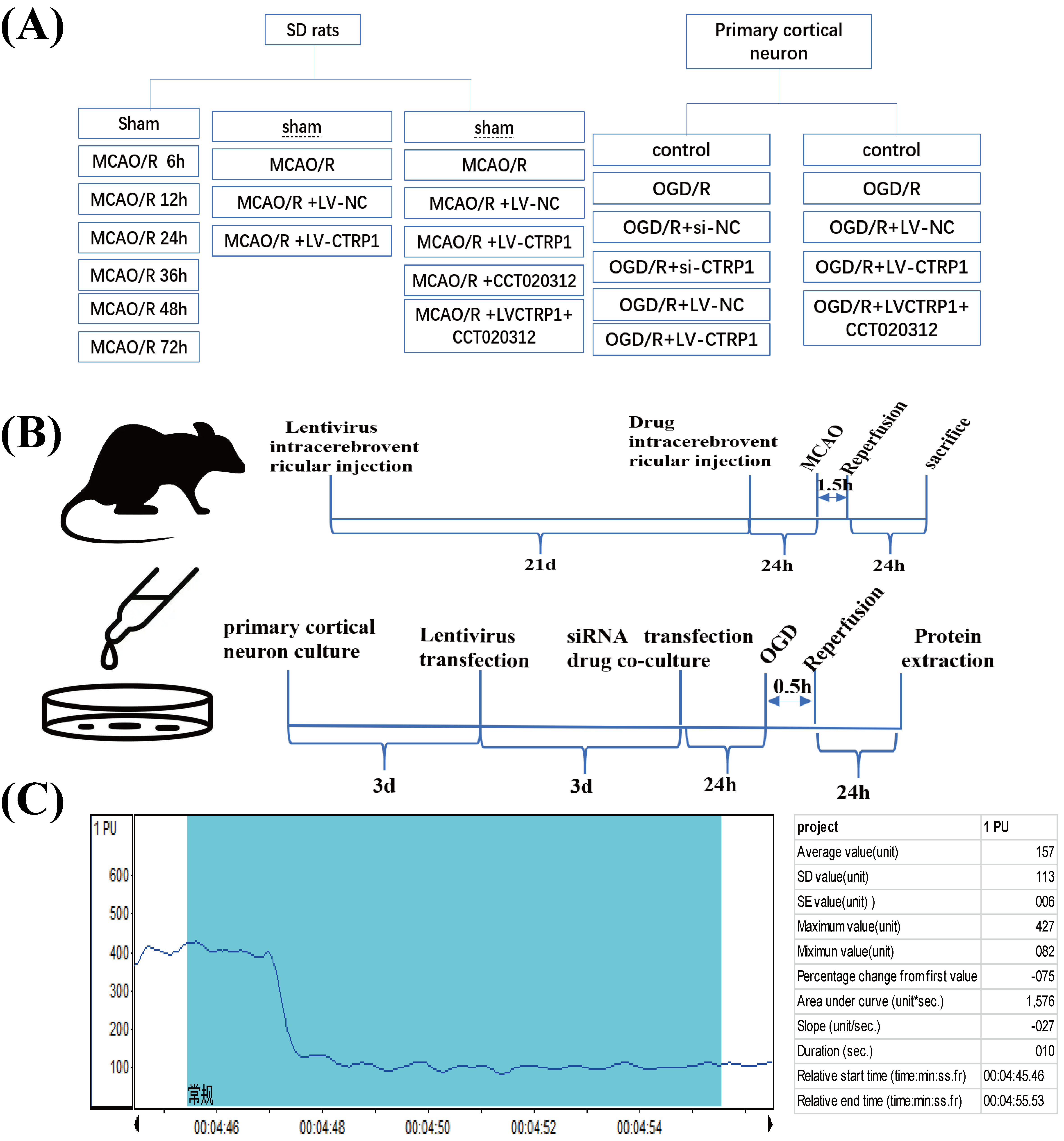

Supplement: Supplementary Figure 1 — Experimental groups and protocol. (A) The groups in the studies. (B) Experimental protocol schedule. (C) Changes in cerebral blood flow were monitored during MCAO by laser Doppler flowmeter. [file Image_1.tif]

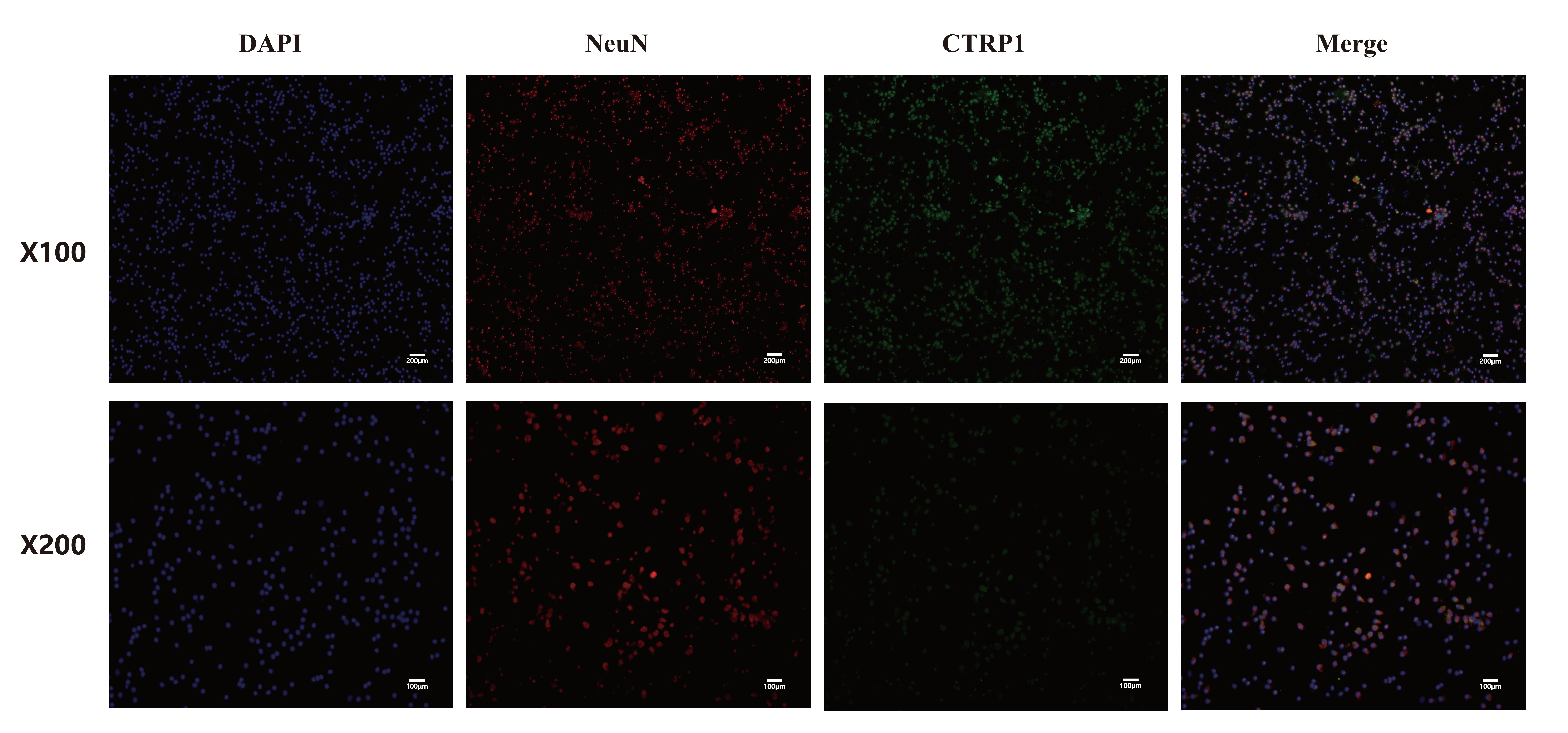

Supplement: Supplementary Figure 2 — The identification of neurons and the expression of CTRP1 in primary cortical neurons (n = 3). The representative images were acquired under × 100 magnification, scale bars = 200 μm, × 200 magnification, scale bars = 100 μm. [file Image_2.TIF]
